# Enhanced In Situ Availability of *Aphanizomenon Flos-Aquae* Constituents Entrapped in Buccal Films for the Treatment of Oxidative Stress-Related Oral Diseases: Biomechanical Characterization and In Vitro/Ex Vivo Evaluation

**DOI:** 10.3390/pharmaceutics11010035

**Published:** 2019-01-17

**Authors:** Viviana De Caro, Denise Murgia, Francesco Seidita, Emanuela Bologna, Gioacchino Alotta, Massimiliano Zingales, Giuseppina Campisi

**Affiliations:** 1 Department of Biological, Chemical and Pharmaceutical Sciences and Technologies, University of Palermo, 90123 Palermo, Italy; denise.murgia@unipa.it; 2 Department of Surgical, Oncological and Oral Sciences, University of Palermo, 90127 Palermo, Italy; francesco.seidita@unipa.it (F.S.); giuseppina.campisi@unipa.it (G.C.); 3 Department of Engineering, University of Palermo, 90128 Palermo, Italy; emanuela.bologna@unipa.it (E.B.); gioacchino.alotta@unipa.it (G.A.); massimiliano.zingales@unipa.it (M.Z.)

**Keywords:** buccal film, *Aphanizomenon flos-aquae*, Eudragit E100, OS-related oral diseases, high frequency homogenization, biomechanical test, ex vivo permeation

## Abstract

In recent years, the key role of oxidative stress in pathogenesis of oral diseases has been emphasized and the use of antioxidant agents has been encouraged. *Aphanizomenon flos-aquae* (AFA) is a unicellular blue-green alga with antioxidant and anti-inflammatory properties. The aim of this study was the formulation and characterization of mucoadhesive thin layer films loaded with AFA, finalized to the treatment of oxidative stress (OS)-related oral diseases. First, to enhance the bioavailability of AFA constituents, the raw food grade material was appropriately treated by a high frequency homogenization able to disrupt cell walls. Thus, Eudragit^®^ E100-based buccal films were produced by the solvent casting method, containing 7% and 18% of AFA. The films, characterized by uniformity in thickness, weight, and drug content, showed low swelling degree, good muco-adhesiveness and controlled drug release. The mechanical tests showed elastic moduli of films of almost 5 MPa that is well-suitable for human buccal applications without discomfort, besides biaxial tests highlighted a marked material isotropy. Permeation studies through porcine mucosae demonstrated the ability of films to promote AFA penetration in the tissues, and when sublingually administered, they produced a drug flux up to six-fold higher than an AFA solution. The new formulations represent an interesting alternative for the development of cosmetics and nutraceuticals with a functional appeal containing plant extracts.

## 1. Introduction

During recent years, there is a rise of oral diseases related to oxidative stress [1]. Oxidative stress (OS) is defined as an imbalance between the amount of reactive oxygen species (ROS) within the cell and the ability of the cell itself to remove these reactive peroxides and free radicals [2].

Among all oral diseases, periodontal disease, oral lichen planus (OLP), and oral cancer are the mostly related to oxidative stress. In particular, OS is associated with the progression of periodontal diseases, causing a disturbance in the regulation of the inflammatory response to the infection [3]. Moreover, it is shown that OS is involved in the, not yet understood, pathogenesis of OLP [4]; higher levels of salivary ROS, lipid peroxidation, nitric oxide, and nitrite levels are found in OLP patients [5]. Furthermore, the antioxidant activity is decreased in OLP patients, compared to healthy subjects [6]. OS is also related with oral cancer: increase in lipid peroxidation and a reduced antioxidant activity are reported [7].

Interestingly, other oral diseases are related with the OS: Dental caries [8], recurrent aphtous stomatitis [2,9], oral sub-mucous fibrosis associated to betel chewing [10], and even to bisphosphonates-related osteonecrosis of the jaws [11].

Considering the influence of the oxidative stress on oral diseases, the use of antioxidant agents should be evaluated. The use of topical antioxidant is widely reported in the therapy of the photo-damaged skin, radio-dermatitis, and in skin rejuvenation [12,13,14]. Topical antioxidant therapy is also reported as a useful treatment of oral pathologies [15]. Antioxidants for local drug delivery in oral mucosa has been used in the treatment of radiation-induced oral mucositis [16], oral lichen planus [17], and in gingival epithelium regeneration [18].

Frequently, antioxidants are extracted from natural products. In particular, the Cyanobacterium *Aphanizomenon flos-aquae* (AFA) has attracted researchers’ interest due to its enriched content in phycocyanin (PCs) and phycocianobilines (PCBs), photosynthetic pigments showing strong antioxidant activity [19].

AFA is a filamentous and heterocytic cyanobacterium, commonly found in nutrient-rich freshwaters as one of the dominant species in cyanobacterial bloom [20]. AFA is also called “Klamath algae”, due to its notably growth in the Upper Klamath Lake in Oregon State, USA, and the no longer used term “blue-green algae”, associated to the Cyanobacteria phylum.

AFA contains numerous compounds, which may be responsible for various health benefits. In particular, a fraction referred as “Immolina” was found to increase the gene expression for chemotactic cytokines that stimulate the leuckocytes migration in tissues [21,22]. In general, the consumption of AFA is associated with an increase of immune system mobilization [23]. As previously reported, AFA is a rich source of PCs, that have demonstrated antioxidant and radical scavenging properties in both in vivo and in vitro models [24,25,26]; this natural compound is considered a strongly anti-inflammatory [27,28]. The PC typical of AFA has a peculiar structure with respect to other microalgae [29], and this seems to explain its significantly higher antioxidant power, which in vitro has shown to be up to 200 times more effective than other PCs [30]. AFA-PCs are reported to be neuroprotectants and showed positive effects on psychological stress and on menopausal well-being [31,32]. AFA also contains mycosporine-like amino acids (MAAs) [33], compounds with UV-absorbing properties and antioxidant activities [34,35]. The latest literature has cast doubt on the safety use of food supplements based on AFA, due to the possibility that these are contaminated by cyanotoxins and in particular by Microcystins (MC) [36]. This alarmism is based on the provisional guideline of 1 μg/L established by the World Health Organization (WHO) on microcystin levels in water [37], which was translated by the Oregon government as 1 μg/g of Klamath Aphanizomenon flos aquae microalgae. Despite this, it could be a mistake to automatically apply standards that are proper for water to cyanobacterial supplements [38], waiting WHO emits clear guidelines on the limits to be imposed for food supplements, it is important to require that each cyanobacterial supplement be tested individually and certified in the content of MC ≤ 1 μg/g. On the other hand, in 1999, the Italian Istituto Superiore di Sanità, tested microcystins-containing Klamath AFA algae on mice for almost one year in different concentrations, and established, as officially reported in the final decision, that the product is safe and suited for human consumption (Ufficio del G.I.P. di Urbino, Decreto di Archiviazione del procedimento, 29 Ottobre 1999) [38].

Considering the beneficial anti-oxidative effects of AFA, this compound could be suitable for the treatment of the oral diseases related to OS. This hypothesis leads us to the realization of a topical delivery system of AFA to apply on buccal mucosa since it provides an excellent opportunity to deliver actives for the loco-regional treatment of oral lesions and pathologies [39].

Moreover, bioadhesive thin layer films for delivering actives into oral cavity have been developed great potential in recent years, as they are identified as an alternative approach to conventional dosage forms [40]. Among various dosage forms, films are a versatile platform for drug delivery capable of guaranteeing in situ adequate concentrations for a long time, increasing the efficiency of drug permeation [41]. Films are easily managed, self-administrable, and fast dissolving dosage forms able to be hydrated by soaking saliva followed by disintegration and/or dissolution releasing active agents. If properly formulated, they can dissolve slowly so to act as a protective dressing of mucosal lesions.

The aim of this study is the formulation and characterization of a drug delivery system of AFA, in the form of mucoadhesive thin film, finalized to the treatment of OS-related oral diseases. The formulation was designed in order to optimize the biomechanical and adhesive properties useful for application in oral cavity and to promote the bioavailability of AFA.

## 2. Materials and Methods

### 2.1. Materials

Aphanizomenon Flos-Aquae powder (AFA) certified in MC content ≤ 1 μg/g was kindly supplied by A.C.E.F S.p.a. (Fiorenzuola d’Arda, Italy). 2,2-Diphenyl-1-picrylhydrazyl (DPPH) was purchased from Sigma-Aldrich (Milan, Italy). Polyvinylpyrrolidone (PVP-K90) and Citric acid was purchased from Fagron Italia S.r.l. (Bologna, Italy). Eudragit^®^ E100 was kindly supplied by Rofarma Italia s.r.l. (Milan, Italy). Urea, Propylene Glycol, Sorbitol and Sodium Saccharin were purchased from Farmalabor (Canosa di Puglia, Italia). Tangerine essential oil was kindly supplied by Agrumaria Corleone S.p.a. (Palermo, Italy).

Non-enzymatic artificial plasma pH 7.4 (PBS) was prepared by dissolving 2.80 g of KH_2_PO_4_ and 20.5 g of Na_2_HPO_4_ in 1 L of distilled water.

Buffer pH 6.8 solution simulating saliva was prepared using NaCl (0.126 g), KCl (0.964 g), KSCN (0.189 g), KH_2_PO_4_ (0.655 g), Urea (0.200 g), Na_2_SO_4_ 10H_2_O (0.732 g), NH_4_Cl (0.178 g), CaCl_2_·2H_2_O (0.228 g), and NaHCO_3_ (0.631 g) in distilled water, according to Gal et al. [42]. 0.9% saline solution was prepared by dissolving 9 g of NaCl in 1 L of distilled water.

All chemicals and solvents of analytical grade were purchased from VWR International (Milano, Italia) and used without further purification. Porcine mucosae were kindly supplied by the Municipal Slaughterhouse of Villabate (Palermo, Italy).

### 2.2. Preparation and Evaluation of AFA Solutions

#### 2.2.1. Preliminary Treatments

AFA (100 mg) was added to 40 mL of methanol 70% *v*/*v* or ethanol 70% *v*/*v*, obtaining, respectively, dispersions M and E. Hence, the dispersions were treated with three different methods. The first method consisted of a treatment in an ultrasonic bath (Branson B 1200 cleaner, Branson Ultrasonic Corporation, Danbury, CT, USA) at 40 kHz for 15 min. The obtained dispersions, named 1-M and 1-E, were transferred in a 50 mL flask and brought to volume with the proper solvent.

In the second method, the solutions were treated with an ultrasonic homogenizer (SONOPULS HD 2070, Bandelin, Germany) at 20 kHz for 10 min, with a pulsated method (0.7 s of activity and 0.3 s of inactivity), maintained the samples in an ice bath during the sonication. The solutions, named 2-E and 2-M, were transferred in a 50 mL flask and brought to volume with the proper solvent. The third method was the same as the second, but the two obtained solutions, named 3-E and 3-M, were vacuum filtered using a 0.45 µm polypropylene membrane (GH polypro-PALL), transferred in a 50 mL flask and brought to volume with the proper solvent.

#### 2.2.2. DPPH Radical Scavenging Assay

To compare the anti-oxidant activity of different AFA extracts the radical scavenging activity of solutions 1–3 M and 1–3 E were measured using the free radical α,α-diphenyl-b-picrylhydrazyl (DPPH) test [43]. The DPPH calibration curve was performed spectrophotometrically (UV/Vis, mod. Pharma Spec 1700, Shimadzu, Tokyo, Japan) in the concentration range of 0.039–0.0156 mg/mL (λ_max_ 515 nm, regression equation: Abs = 2.278 + 3.029 × [DPPH mg/mL], R = 0.999). Aliquot of 2.5 mL of 2 mg/mL AFA solution (1-M, 1-E, 2-M, 2-E, 3-M, 3-E) previously described were transferred in disposable polystyrene cuvettes and added to 250 µL DPPH in an ethanol solution (0.0295 mg/mL).

The DPPH concentration has been measured at λ = 515 nm every 15 min up to 120 min in the range of 400–800 nm.

#### 2.2.3. Qualitative Analysis of Extracts

To evaluate any differences between the extracts obtained with the two solvents, a qualitative HPLC-DAD analysis was carried out using a method described in the “AFA assay” paragraph.

### 2.3. AFA Films Preparation

Mucoadhesive films of AFA have been prepared by the solvent casting method. Eudragit^®^ E100 (650 mg) and Citric acid (100 mg) were added to ethanol (10 mL) and water (2 mL) and stirred until complete dissolution. Then, PVP K90 (190 mg), urea (100 mg), sorbitol (60 mg), sodium saccharin (30 mg), and propylene glycol (210 mg) were added to the solution.

Separately, AFA (100 mg or 300 mg) in 20 mL ethanol was ultrasonic homogenized (SONOPULS HD 2070, Bandelin) for 10 min, with a pulsated method (0.7 s of activity and 0.3 s of inactivity), maintaining the sample in an ice bath during the sonication. When a film containing 300 mg of AFA was prepared (AFA300), the homogenized AFA solution was vacuum filtered (GH polypro PALL, 0.45 µm) to remove the insolubilized residues of cyanobacteria cell wall.

Finally, the two solutions were mixed and again sonicated in the same conditions for 10 min. During the final 30 s of treatment, 50 µL of Tangerine essential oil and Tween 20 in ethanol solution was added.

The mixture was poured into a silicon mold having an area of 20.25 cm^2^ and dried in an oven (StabiliTherm, Thermo Scientific) at 35 °C for 24 h.

The so-formed films (AFA100 and AFA300, containing for patch 100 mg and 300 mg of algae, respectively) was then left equilibrate at room temperature and humidity for 24 h, checked for any imperfections or air bubbles and cut by biopsy punches. The samples were put in polystyrene disposable weighing boats, packed in polyethylene heat-sealed bags, and stored at room temperature to maintain the integrity and elasticity of films.

### 2.4. Film Weight, Thickness and Drug Load Uniformity

Three disks 3.6 mm in diameter from each batch randomly selected were weighted and measured in diameter and thickness with a vernier caliper and a digital micrometer (VWR International, Milano, Italy), respectively. Each disk was then transferred into 10 mL flask, brought to volume with methanol and sonicated to solubilize both the drug and excipients. The amount of AFA released from films was measured spectrophotometrically at λ = 334 nm and 664 nm using the appropriate calibration curves and blank. At the concentrations used, dissolved excipients and polymers does not interfere with the UV absorption of AFA.

The uniformity of batches was evaluated calculating the average and standard deviation for all the considered parameters on three disks for batch.

### 2.5. Surface pH of Film

Randomly selected film disks were put on the surface of an agar plate, prepared by dissolving 2% (*w*/*v*) agar in warmed simulated saliva (pH 6.8) under stirring, and then pouring the solution into a Petri dish until it gelled at room temperature. After addition of 0.5 mL of simulated saliva on the disk, it was left to swell for 2 h and the pH was measured using a pH meter (HI 2211 pH/ORP Meter, Hanna Instrument, Woonsocket, RI, USA) by placing pH probe in close contact with the wetted film surface. The experiment was performed in triplicate for each different film composition.

### 2.6. Mechanical Tests

Two kinds of experimental set-ups have been considered on the AFA films: A uniaxial displacement-controlled monotone traction test conducted by a Bose Biodynamics 200; a plane controlled strain monotone test conducted by a Bose^®^ ElectroForce^®^ Planar Biaxial TestBench Test Instrument, both located at the Bio/Nano Mechanics for the Medical Sciences Laboratory at AteN Center of Palermo University.

The experimental campaign has involved 24 samples, twelve for each film, namely AFA100 and AFA300. They have been grouped in six sets, three for AFA100 and three for AFA300 composition. One of the groups of the AFA100 and the AFA300 have been tested in uniaxial conditions along a direction of the edge of the sample. The other group of the AFA100 and of the AFA300 have been tested in the orthogonal direction in order to investigate the direction-dependent mechanical behavior, as well as to check the order of magnitude of the measures. The third group of specimens for each composition was loaded in biaxial conditions to investigate material isotropy and the effect of load combination.

#### 2.6.1. Uniaxial Tests

The uniaxial traction tests have been conducted with a specific protocol involving the preparation of four rectangular strips normalized to avoid possible scale effects in the evaluation of strength and moduli. The samples have dimensions of 0.5 mm width, length 9.82 mm length, and 0.45 mm thickness for each direction parallels to the main edge of the sample. Samples were placed between two clamps of the BOSE Biodynamics 200 equipped with a load cell of 220 N. The test has been conducted in displacement control, with a rate of 0.1 mm/s and final displacements of 5 mm.

The two groups of samples obtained by the planar films have been prepared by trimming the film along two orthogonal directions, longitudinal and transverse direction, dubbed in the following x_1_ and x_2_, respectively, with an overall number of 4 samples for each direction.

Loads and displacements were measured continuously during the test by the WinTest software (TA Instruments, New Castle, PA, USA) that is the control software of the testing equipment allowing for the estimation of the Young modulus and the material strength.

The Young modulus of the material is the slope at the origin of the stress-strain curve obtained in the tensile test, in terms of the engineering measures of stress *σ* and strain *ε*, with a dimension in the SI system of MPa and %, respectively.

The engineering measures of stress and strains have been estimated by the WinTest by means of the classical definitions:

(1) $$\sigma \left[\mathrm{MPa}\right]=\frac{F\left[N\right]}{A\left[{\mathrm{mm}}^{2}\right]}$$

(2) $$\varepsilon \left[\%\right]=\frac{l_{f}-l_{0}}{l_{0}}=\frac{l_{f}}{l_{0}}-1$$

where *σ* indicates the tensile stress, *F* the load on sample and expressed in N, *A* the cross-section area, expressed in mm^2^, *l_f_* the final length of the sample measured between the clamps, and *l*_0_ is the distance between the clamps. The young modulus has been measured as the ratio among the first recorded stress and the first recorded strain [44,45]. This procedure was conducted for both x_1_ and x_2_ directions of the film.

#### 2.6.2. Biaxial Tests

The test involves the application of orthogonal displacements (forces) on a planar specimen of the material. The used testing equipment involves four engines that operate independently with load cells mounted on two orthogonal directions.

The specimens have been standardized by cutting samples of 1 cm × 1 cm and 0.45 mm of thickness and were held from each side by a system of hooks and nylon threads.

The mechanical test requires accurate measures of the planar strain tensor of the plane specimen requiring the knowledge of the axial strains along the orthogonal directions, namely *ε*_1_ and *ε*_2_, and the shear strain *ν*_12_ = 0 in presence of isotropy. The strain field is measured by means of a digital video estensometer (DVE) that requires the presence of five black markers on the sample monitoring that allows for image correlation elaborated by the wintest software that controls the testing equipment. The set-up instrument was in control of displacement, 5 mm for each side, and 0.1 mm/sec as rate of deformation. The elastic modules in two direction was calculated considering a plane stress for biaxial test as:

(3) $$\left\{\begin{array}{c} \varepsilon_{1}\\ \begin{array}{l} \varepsilon_{2}\\ \varepsilon_{3} \end{array} \end{array}\right\}=\left[\begin{array}{ccc} \frac{1}{E_{1}} & -\frac{\upsilon_{21}}{E_{2}} & -\frac{\upsilon_{21}}{E_{2}}\\ -\frac{\upsilon_{12}}{E_{1}} & \frac{1}{E_{2}} & -\frac{\upsilon_{21}}{E_{2}}\\ -\frac{\upsilon_{21}}{E_{2}} & -\frac{\upsilon_{21}}{E_{2}} & \frac{1}{E_{1}} \end{array}\right]\left\{\begin{array}{c} \sigma_{1}\\ \begin{array}{l} \sigma_{2}\\ 0 \end{array} \end{array}\right\}$$

where $\nu_{ij}=-\varepsilon_{j}/\varepsilon_{i}$ is the Poisson coeffients for load in the i-direction and $\nu_{ji}=-\varepsilon_{i}/\varepsilon_{j}$ is the Poisson coefficient for load in the j-directions. Poisson coefficients characterized the material in case of anisotropy. The symmetry of the matrix coefficients in Equations (1) and (2) requires that $\upsilon_{12}/E_{1}=\upsilon_{21}/E_{2}$. The small thickness of the specimen allows for the assumption $\varepsilon_{3}≅0$.

### 2.7. Ex-Vivo Mucoadhesion Strength Measurement

The ex-vivo mucoadhesive strength evaluation of prepared films was performed by the modified two-armed physical balance method [46]. Porcine buccal mucosa excised from just slaughtered pigs was used as model tissue and handled without any pre-treatment. A tissue portion of the inner part of the cheek was glued by cyanoacrylate resin (Super Attak Loctite^®^, Henkel Italia Srl, Milan, Italy) on a glass support (Petri dish) and placed in a thermostatic bath at 37 ± 1°C. The film’s disk was fixed using a bi-adhesive to the lower side of a rubber stopper hanging from the balance arm. Before starting the measurements, the mucosal tissue was wetted with 50 µL of simulated salivary fluid and then the film was placed on the tissues so it just touched the mucosal surface and a light force with a fingertip was applied for 20 s. A temperature of 37 ± 1 °C was maintained throughout the experiment. The measurements started 5, 10, and 15 min after application, thus allowing for different time contacts. 0.8 cm diameter disks, taken from 3 different films, were used to perform the test.

The grams required to detach the film from the mucosal surface provided the measurement of mucoadhesive strength, according to the equation:

Force of adhesion (N) = (g × 9.81)/1000

Then, detachment force was calculated as:

Detachment force (N/m^2^) = Force of adhesion (N)/Surface area (m^2^)

The maximum adhesive force was determined as the average of 3 measurements (n = 3) [39].

### 2.8. Swelling Test

The water uptake was quantified gravimetrically. A dry film disk (surface 0.5 cm^2^) was placed on a glass support into an analytical balance and weighed. Then, every 5 min, up to complete dissolution of film, 0.1 mL of artificial saliva pH 6.8 were added on the disk. At every time interval, after removal of the excess water surface by light blotting with a filter paper the weight of the wet disk was assessed. The test was performed on one disk of six different batches.

Results were reported as means (±SE) of 6 disks from different batches of film. The swelling Index (SI) was calculated using the following equation:

$$Swelling\;Index=\;\frac{W_{0}+\left(W_{t}-W_{0}\right)}{W_{0}}$$

where *W_t_* is the weight of film at time t, and *W*_0_ is the weight of dry film.

### 2.9. In Vitro Dissolution Test

Film dissolution in buffer solution simulating saliva was assessed using the flow through the system previously described [39]. Briefly, the system consists of beker containing a buffer solution simulating saliva (100 mL) thermostatted at 37 °C from which the liquid is forced to a Plexiglass release chamber by a peristaltic pump (Bio-Rad Econo Pump, Hercules, CA, USA). The flow rate of saliva was maintained constant at 0.5 mL/min. In the chamber, a film disk of 1.1 cm diameter sandwiched between two cellulose acetate membranes to avoid fragments of films clogging tubes was allocated. In the chamber, the salivary layer wetting the sides of the disk was about 0.2 mm thick. The temperature was maintained at 37 ± 0.1 °C by submerging the chamber in a thermostatic bath. Aliquots (1 mL) of solution coming out from the release chamber every 2 min were collected and the drug amount was then quantified by UV detection, using the appropriate blank and calibration curve. Results were averaged on 6 disks from 6 different batches of film. Every experiment was considered until the complete dissolution of the disk occurred and the amount of drug released matched the original drug content of disk. Release data were elaborated using Kaleidagraph v.3.5 (Synergy Software Inc, Reading, PA, USA) as software.

### 2.10. Ex Vivo Permeation of AFA throughout Porcine Mucosa

The permeation of AFA, which is released from the film throughout the porcine sublingual or buccal mucosa, was evaluated using Franz type diffusion cells (Permeagear, flat flange joint, 9-mm orifice diameter, 15-mL acceptor volume, SES GmbH—Analysesysteme, Bechenheim, Germany), used as a two-compartment open model. Mucosal specimens (kindly supplying by Municipal Slaughterhouse of Villabate, Palermo, Italy) consisted of tissue removed from the ventral surface of the tongue (sublingual mucosa) and the vestibular area of the retromolar trigone (buccal mucosa) of freshly slaughtered domestic pigs of 12 months of age. Specimens were prepared, as described previously [47]. Briefly, after sampling, all specimens were immediately placed in a refrigerated transport box and transferred to the laboratory within 1 h. The specimens were surgically treated to remove excesses of connective and adipose portion and some of these were used fresh; the remaining specimens were stored at −40 °C for periods up to six months. At the time of use the specimens were equilibrated at room temperature and dipped for 30 s in saline solution previously warmed to 60 °C; the connective tissue was then carefully peeled off from the mucosa (slides 250 ± 25 μm and 100 ± 25 μm thick for buccal and sublingual tissues, respectively) to obtain the heat-separated epithelium along with the intact basal lamina [48]. The thickness was measured using a digital micrometer. The heath treatment does not involve permeability and/or integrity modifications in porcine buccal mucosa [47]. Before the start of experiments, specimens were equilibrated in PBS for about 3 h at room temperature to remove biological matter, which could interfere with drug analyses. The equilibration medium was replaced with fresh PBS every 15 min.

Once the membrane has been mounted in the Franz diffusion cells the donor chamber it was equilibrated for 30 min at 37 ± 0.1 °C adding PBS in both the donor and the acceptor compartment. This step was followed by the removal of PBS from the donor compartment and replacement with 1 mL of AFA solution in simulated saliva (15 mg/mL) or one AFA film’s disk (8 mm diameter) in 0.4 mL of simulated saliva applied to the apical side of the membrane. At regular time intervals (15 min for sublingual permeation experiments and 30 min for buccal ones), samples (0.5 mL) were withdrawn from the acceptor compartment and the sample volume was taken out and replaced with fresh fluid.

The permeation experiments using buccal mucosa were carried out for 4 h, whereas for 2 h using sublingual ones. The AFA amount in the acceptor chamber was quantitatively determined by UV detection using the appropriate blank and calibration curve. The analyses were performed on 2 disks of six different batches. Results were reported as means ±SE (n = 12). At the end of experiments, mucosal integrity was checked, as previously described [49].

The flux values (*Js*) across the membranes were calculated at the steady state per unit area by linear regression analysis of permeation data, following the relationship *Js* = Q/At (mg/cm^2^·h), where Q is the amount of AFA recovered in the acceptor compartment, A is the cross-sectional area available for diffusion (0.636 cm^2^), and t is the time of exposure (h). Data were elaborated using Kaleidagraph 3.5 (Sinergy Software Inc, Reading, PA) as software. Analysis of variance was followed by *t*-test. The level of significance was selected as *p* < 0.05.

#### Quantification of AFA Entrapped into the Porcine Mucosae

At the end of each experiment, the residual AFA amount entrapped into the mucosal tissue was quantified by extraction. Each buccal mucosa specimen was washed with PBS (3 × 2 mL) and was then dipped for 5 min in warmed (50 °C) methanol (1.5 mL). The extraction was repeated three times and the collected mother liquors were quantitatively transferred in a 5 mL flask, and brought to volume. The amount of AFA extracted was evaluated by UV analysis using the appropriate calibration curve and blank. The same extraction treatment was also performed on mucosal specimens subjected to experimental phase in the absence of drugs and was used as control.

### 2.11. AFA assay

#### 2.11.1. UV-Vis Analysis

The amount of AFA entrapped into the films was measured spectrophotometrically using the appropriate calibration curve and blank (UV/VIS mod. Pharma Spec 1700, Shimadzu, Tokyo, Japan). UV method was found simple, accurate, and reproducible. A stock solution of AFA (0.5 mg/mL) in ethanol 70% *v*/*v* was ultrasonic homogenized for 10 min, with a pulsated method (0.7 s of activity and 0.3 s of inactivity), then diluted working standards were freshly prepared in the proper solvent before analysis. AFA solutions show three main absorption peaks at 334, 408 and 664 nm. Validation parameters in methanol: λ_max_ = 334 nm, linearity range 0.02–0.1 mg/mL, E_1%_ = 0.232, regression equation Abs = 0.00353 + 1.966x (mg/mL), R = 0.996, standard error 0.00677; λ_max_ = 408 nm, linearity range 0.005–0.1 mg/mL, E_1%_ = 0.00725, regression equation Abs = 0.00301 + 0.4240x (mg/mL); R = 0.961, standard error 0.00406 and λ _max_ = 664 nm, linearity range 0.005–0.1 mg/mL, E_1%_ = 0.00231, regression equation Abs = 0.00101 + 0.1230x (mg/mL); R = 0.969, and standard error 0.00103.

At the testing concentrations, no interferences between AFA and components of formulations were observed and no change in the absorbance of the drug at its λ_max_ was experienced when AFA solutions were analyzed in presence of excipients. In analogy, the amount of AFA entrapped into the membrane was measured after extraction from mucosal tissue by methanol.

A calibration curve in artificial saliva pH 6.8 was also constructed for AFA quantification during the dissolution test. Validation parameters: λ_max_ = 334 nm, range 0.06–0.6 mg/mL, E_1%_ = 0.295, regression equation Abs = 0.02793 + 1.6188x (mg/mL), R = 0.999, and standard error 0.01627.

The amount of AFA transferred in the acceptor compartment during permeation test was measured in phosphate buffer solution (pH 7.4) using the appropriate calibration curve and blank. Validation parameters: λ_max_ = 334 nm, linearity range 0.005–0.1 mg/mL, E_1%_ = 0.279, regression equation Abs = 0.00510 + 3.2991x (mg/mL), R = 0.995, and standard error 0.01133.

Intraday and interday variations, observed during collection of experimental data, were lower than sensibility.

#### 2.11.2. HPLC Analysis

HPLC analyses were performed with a HPLC Shimadzu LC-10AD VP instrument (Tokyo, Japan) equipped with a binary pump LC-10AD VP, a UV SPD-M20A Diode Array detector, a 20 μL injector and a computer integrating apparatus (EZ Start 7.4 software, Shimadzu Scientific Instruments, Inc., Columbia, MD, USA). Chromatographic separation was achieved on a reversed-phase column SeQuant^®^ Zic^®^-Hilic, (5 μm, 200 Å, 150 × 2.1 mm, Merck, Germany), a mobile phase consisted of acetate buffer 5 mM pH 6.5 (A) and Acetonitrile (B). For separation of AFA components the gradient method was developed as follows: A:B (0.5:99.5→0.01–5.00 min, 40:60→5.00–11.00 min; 40:60→11.00–30.00 min). The flow rate was set at 0.3 mL/min, the UV wavelength range 200–700 nm and set at 260, 334, 407, 665 nm to identification.

### 2.12. Stability Tests

In order to evaluate the stability of AFA films over time, tests were conducted on AFA100 and AFA300 films packed in polyethylene bags heat-sealed and kept protected from light and room temperature for six months.

#### 2.12.1. Attenuated Total Reflectance (ATR)

AFA100 and AFA300 films freshly prepared and six-month-old were analyzed by ATR-FTIR and compared with spectra of empty film and AFA raw material. Spectra were recorded on a Fourier Transform Infrared Spectrometer (FTIR) Bruker Alpha (Bruker, Billerica, MA, USA) equipped with an ATR unit plug-and-play having diamond crystal for surface analysis and computer integrating apparatus (Opus spectroscopy software 7.5, Bruker). Spectra were obtained by accumulation of 32 scans between 4000 and 450 cm^−1^, at 4.0 cm^−1^ resolution and rationed to the appropriate background spectra.

#### 2.12.2. In Vitro Dissolution Test

To evaluate any possible release alteration induced by the storage time, in vitro dissolution studies were carried out on six-month-old AFA100 and AFA300 films as described in 2.8 paragraph. 

The test was repeated three times for each formulation.

## 3. Results and Discussion

### 3.1. Preliminary AFA Treatments

AFA widely used in food-integrators is commercially available as powder (food-grade) obtained by freeze-drying of freshly harvested microalga with cell matrix unaltered. This source could represent an obstacle to bioavailability of antioxidant components because, to be absorbed, they must be released from the cells in which they are entrapped in the dry state. Additionally, for this reason, there are numerous patents on the extraction of AFA constituents and their applications in the literature, demonstrating the great interest for this algae and its properties [50,51].

Therefore, the first objective was the use of raw food grade material, appropriately treated by a homogenization process able to disrupt the cellular matrix to enhance the bioavailability of its constituents. For this reason, two treatments of AFA solutions were proposed: Sonication in ultrasonic bath (1-treatment), and a high frequency ultrasonic homogenization (2-treatment). The high-frequency homogenized solutions were also filtered (3-treatment) and tested, in order to assess the contribution of insoluble cellular residue on antioxidant capacity of the extract. The used solvents were aqueous Methanol (70% *v*/*v*; M solutions) and aqueous Ethanol (70% *v*/*v*; E solutions). As a consequence, six solutions (1-M, 1-E, 2-M, 2-E, 3-M, and 3-E) were obtained and evaluated in term of antioxidant capacity.

Solutions 1-M and 1-E, treated in ultrasonic bath, appeared brown, with an intense opalescence showing sedimentation of powdery material. Solutions 2-M and 2-E, treated by ultrasonic homogenization that allows cellular lysis, appeared green-brown, slightly opalescent and with an easily dispersible sediment, more evident in 2-M solution. Some of these were filtered with a hydrophilic polypropylene membrane (Millipore, Type HA, 0.45 µm) obtaining 3-M and 3-E solutions.

**Figure 1:**
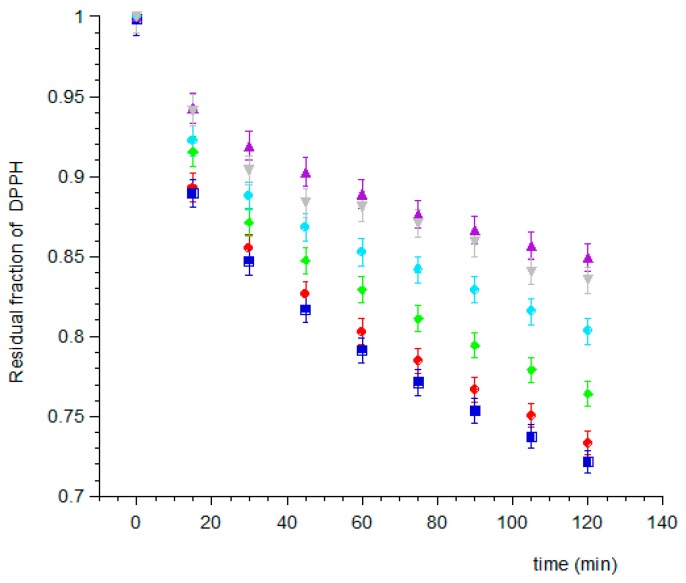
Residual DPPH concentration as a function of time in the different samples of treated AFA solutions: (▼) Methanol in ultrasound bath; (⬤) Methanol in homogenizer; (▲) Methanol in homogenizer and filtered; (◆) Ethanol in ultrasound bath; (⏹) Ethanol in homogenizer; and (◆) Ethanol in homogenizer and filtered.

To determine the antioxidant power of obtained solutions the DPPH Test was used. Therefore, six samples were spectrophotometrically analyzed every 15 min for 2 h (Figure 1) to evaluate the reaction rate of each extract when added to DPPH.

The results showed that the ethanol AFA solutions have higher anti-oxidant activity, compared with the methanol ones. This is probably due to the high hydro-solubility of most of AFA components. Moreover, methanol could induce a partial proteins denaturation, leading to a decrease in anti-oxidant concentration, as the MAAs.

Regarding the type of pre-treatment, the results show that the solutions treated with ultrasonic homogenization (types 2 and 3) have higher antioxidant capacity, compared with the type 1 solutions. These results demonstrate the high efficacy of the ultrasound homogenizer, which disrupt AFA cell walls. The antioxidant activity of filtered and unfiltered ethanol solutions is comparable, demonstrating that the insoluble residue does not contribute to the antioxidant properties of the algae.

**Figure 2:**
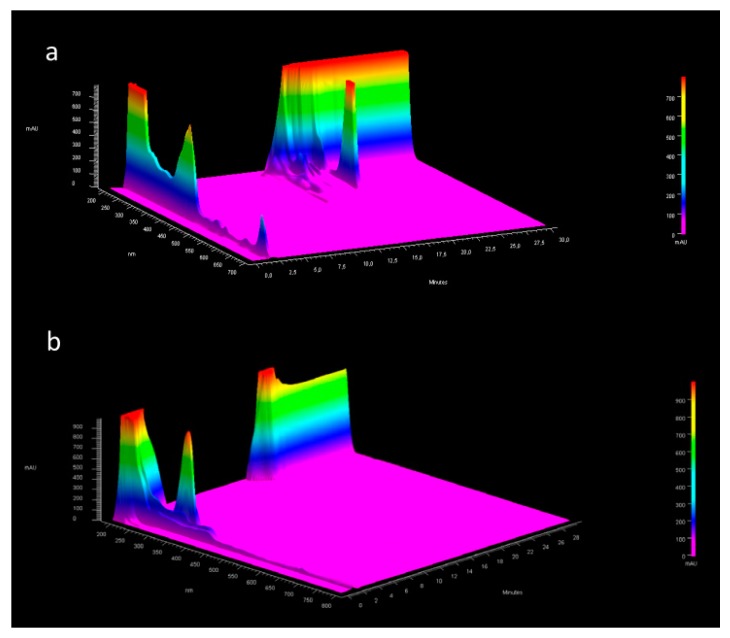
3D image of chromatographic elution of a (**a**) Methanol extract and (**b**) Ethanol extract from 0 to 30 min.

Qualitative studies by HPLC-DAD analysis of extracts were performed to evaluate any differences among the extraction methods. Chromatograms showed pick characteristics at 665, 334, and 260 nm attributable at PCs, MAAs and phenolic compounds, respectively (Figures S2–S4, S6 and S7). Figure 2 (and Figures S1 and S5) reported 3D image of chromatographic elution of the extracts from 0 to 30 min in order to highlight all the separated compounds that have different maximum absorption peaks. It is possible to highlight that the methanol extract shows a peak at Rt 20.47 min attributable to the MAAs (Figure S3), which is absent in the ethanol extract (Figure S7). On the other hands, chromatographic elution of ethanol extract presents two picks at Rt 2.76 and 3.38 min (Figure S7). having UV spectra typical of MAAs. The authors therefore suppose that the choice of extraction solvent affects the chemical stability of the components and this could be the cause of the different anti-oxidant power of the two extracts. Chromatograms for each fixed wavelength are reported in the Supplementary Information File.

Therefore, for preparation of mucoadhesive films, the AFA ethanol solution obtained by high frequency homogenization was chosen.

### 3.2. AFA Films Formulation

One of the most common approaches to control the delivery is to entrap the drug in a matrix system. In particular, the formulation of bioerodible matrix films, in which AFA was homogenously dispersed, has been designed as alternative to the conventional topical drug formulations with the aim of improving AFA retention on oral mucosa, and consequently, the bioavailability of the actives.

The oral films must be manageable and flexible, and at the same time, resistant and stable. Moreover, they must have adequate mucoadhesion properties and a reproducible release pattern. Finally, films should have a good taste to improve palatability and patient’s compliance.

Therefore, a readily water-soluble polymer such as Eudragit^®^ E-100 was chosen for the formulation as matrixing polymer, together with PVP-K90 for conferring mucoadhesive properties. Eudragit^®^ E100 is a biocompatible, mucoadhesive cationic polymer. This polymer in ethanol solution confers on mixture a low viscosity, useful to pour and homogeneously distribute it into the mold, without loss of product. In order to provide homogeneity and clarity to the films, Eudragit^®^ E-100 was acidified by citric acid. Moreover, citric acid facilitates polymer dissolution and acts as a preservative in the final formulation.

PVP-K90 possesses strong chemical bonding affinity with mucins and high chemical and biological inertness. Therefore, PVP has been used in order to achieve an intimate and prolonged contact with oral mucosa, a crucial requirement for an oral Adhesive Drug Delivery System [52]. Flexibility and softness characteristics have been conferred to the films by the introduction of moisturizing agents and plasticizers [53]; in particular, Sorbitol and Propylene Glycol are chosen for their ability to retain water within the film and for their inhibitory activity on bacterial growth, especially when they are in association [54]. Urea was chosen as hydration and permeability barrier function enhancer [55]. To mask the bad taste of algae and make the films palatable, Saccharin was preferred as a sweetening agent, due to its stability in acid environments and Tangerine essential oil, was used as a flavoring agent. The quantitative composition of films has been selected on the basis of our previous experiences [56].

Loaded AFA mucoadhesive films have been prepared by the solvent casting method. The method is simple, inexpensive, and does not imply the use of organic solvents. The method has been the most appropriate to obtain a solid dispersion of drug in a matrix system with a high reproducibility of results. Two formulations, different in AFA content, were prepared: AFA100 using 100 mg of Algae homogenized in ethanol and then mixed with the other components of formulation, and AFA300 using 300 mg of Algae homogenized in ethanol solution and was filtered prior to adding it to the other components. In AFA300 the filtration was needed due to a large amount of cell wall residues that would confer an un-well appearance to the film.

Once the drug solutions and excipients have been mixed and poured in silicon mold, they were dried in oven at 35 °C for 24 h. To establish temperature and time, preliminary tests were conducted; temperatures over 40°C leads to a rapid solvent evaporation and the films presented bubbles and a non-homogeneous surface. A drying protracted over 28 h leads to brittle and stiff films.

**Figure 3:**
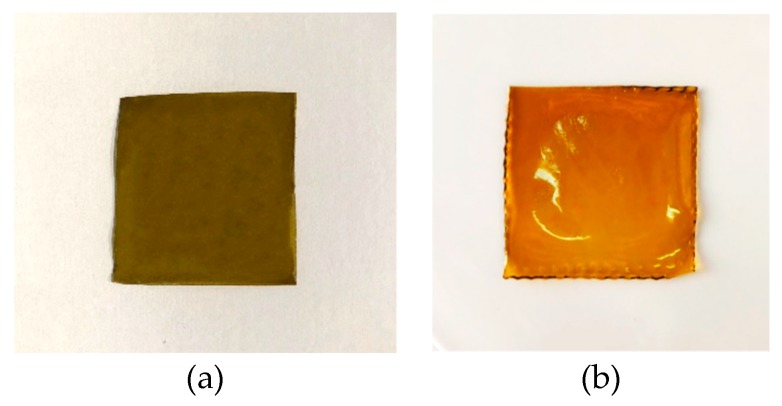
AFA100 (**a**) and AFA300 (**b**) matrix films.

The obtained films (Figure 3) were cut, using a biopsy punch, into disks of 0.4, 0.8, and 1.1 cm diameter, respectively, used for subsequent analyzes. All the disks cut from the same film were considered as belonging to the same batch.

### 3.3. Film Weight, Thickness and Drug Load Uniformity

The reproducibility of films has been assessed by measuring, from three different batches, the average weight, thickness, and drug content of 0.126 cm^2^ disks (n = 3) of the same batch. All data are reported in Table 1 and confirms high product reproducibility. The surface pH of films resulted in a range from 6.31 to 6.55, values similar to the salivary fluid and compatible with the oral cavity, so as to not give any kind of irritation to the mucosal tissue.

### 3.4. Mechanical Tests

A large experimental campaign has been conducted on the films to measure their mechanical properties in terms of [57,58]: (i) Young modulus *E*; (ii) material strength, namely the ultimate stress carried by the material before failure, dubbed *σ*_u_; and (iii) ultimate strain denoted *ε*_u_ in the following, that is the strain undergone by material at the onset of failure. Two kinds of experimental set-ups have been considered on the AFA films: A uniaxial displacement-controlled monotone traction test and a plane controlled strain monotone test.

In the uniaxial test the Young modulus of the material is the slope at the origin of the stress-strain curve obtained in the tensile test, in terms of the engineering measures of stress *σ* and strain *ε*, expressed in MPa and %, respectively. The Young modulus has been expressed as the ratio among the first recorded stress and the first recorded strain [44,45].

**Figure 4:**
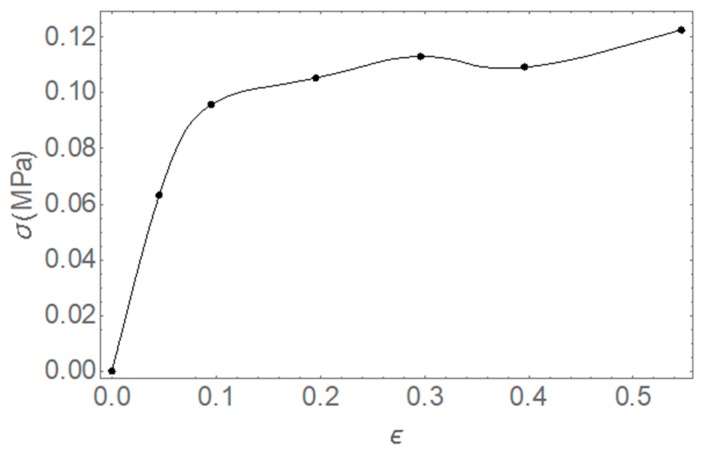
Typical stress-strain curve for uniaxial test on AFA-100.

As representative, the experimental curve of uniaxial mechanical test on AFA100 was reported in Figure 4.

This procedure was conducted for both x_1_ and x_2_ directions of the film and averaged values of the obtained moduli has been reported in Table 2.

The first two columns of the table report the averaged strengths of the material (with ±SD); ultimate strains have been reported in the third and fourth column, whereas the elastic moduli for the two directions x_1_ and x_2_ have been reported in the last two columns of the table.

Since the effects of the different composition of the two films could introduce material’s anisotropy [59], this effect has been investigated, resorting to a biaxial experimental campaign of mechanical tests allowing to capture material anisotropy also in the presence of non-linear measures of strains [60,61]

**Figure 5:**
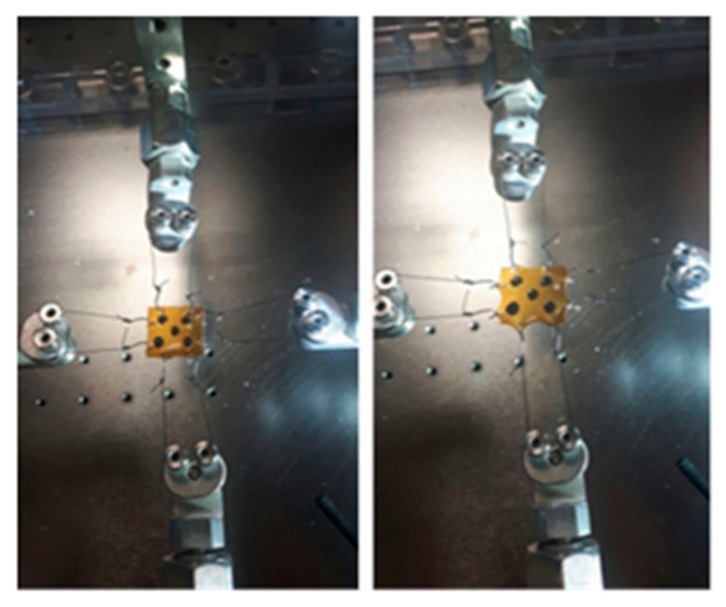
Experimental set up for biaxial test.

**Figure 6:**
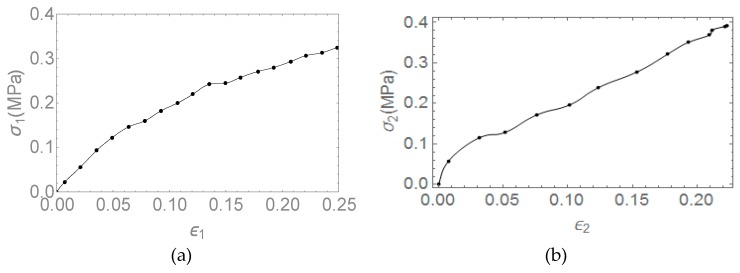
Stress strain curve of AFA100: (**a**) Direction x_1_, and (**b**) direction x_2_.

**Figure 7:**
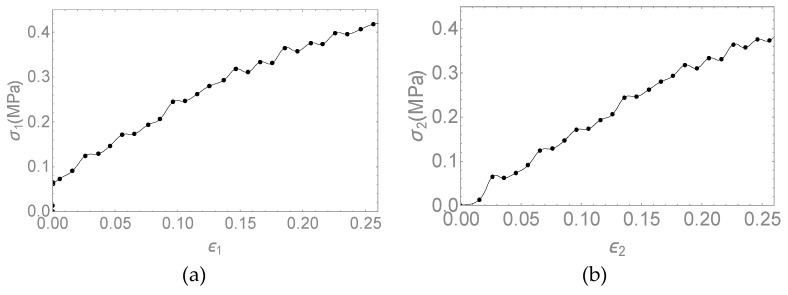
Stress strain curves of AFA300: (**a**) Direction x_1_, and (**b**) direction x_2_.

The averaged values of the elastic moduli along the two directions *E*_1_ and *E*_2_ may be evaluated by the biaxial testing machine. The specimens were held from each side by system of hooks and nylon threads, as shown in Figure 5:

Data obtained from the tests show the loads in direction x_1_ and x_2_ and the displacements in these respective directions yielding, by means of the DVE, and the stress-strain curves, as in Figure 6 and Figure 7.

In the Table 3 averaged moduli *E*_1_ and *E*_2_ and the averaged Poisson coefficient calculated on 4 samples of each film composition have been reported.

Data analysis of the biaxial tests confirms the results of the uniaxial tests, that the material shows an isotropic behavior, as shown by the values of the Young modulus and of the Poisson coefficients that are almost coincident.

The results of the mechanical tests show that the elastic modulus of the films is almost 5 MPa, which is well-suited for human buccal [62] applications without discomfort or local injuries.

Beside elastic moduli, the measurements of the Poisson coefficients highlight a marked material isotropy that is the mechanical behavior, independent of the load direction. Indeed, Poisson coefficients evaluated in condition of plane elasticity assume equal values that means that the material does not possess a sub-structure with a lower-level anisotropy.

Additionally, the experimental campaign provided the material strength that must be considered in production and packaging to maintain intact films. The mechanical investigation may be further extended to show how the mixtures of the constituents alter the mechanical properties and the isotropy of the material and it will be reported elsewhere.

### 3.5. Ex-Vivo Mucoadhesion Strength Measurement

The adhesive properties of the film are fundamental, as long as they influence the ability of the dosage to be retained at the site of action, in contact with the mucosal membrane. Moreover, mucoadhesion is important in order to avoid intraoral detachment and the consequent ingestion. This property of formulations has been studied using a wide variety of methods, which are influenced by instrumental variables and experiment design [63]. The mucoadhesive force of films was measured using an analytical balance modified according to the literature [46]. The results were calculated by the described equations in the Methods Section and reported in Table 4.

The results highlighted that, after 5 min, it had already established bonds between mucins and polymers, and they increased over time, maintaining the formulation well anchored to the mucosa. This phenomenon could be justified, being Eudragit E100 a linear polymer with tertiary amine groups that can easily change its conformational state as a function of pH and the salt composition of the media [64]. At salivary pH 6.8, modifying the electric charge by interaction of its positive groups with negative counterions of mucins (negatively charged) Eudragit promotes mucoadhesion and could also interact with lipid of membranes [64]. Slightly higher values in the force adhesion were observed for AFA100, probably due to contributing in adhesion from cells wall of unfiltered AFA extract.

### 3.6. Swelling Test

The swelling test was carried out in order to measure the capability of film to attract biological fluids increasing its volume. For a buccal film, the swelling should be minimal in order to avoid the discomfort to the patient for the presence of an extraneous body in the oral cavity.

**Figure 8:**
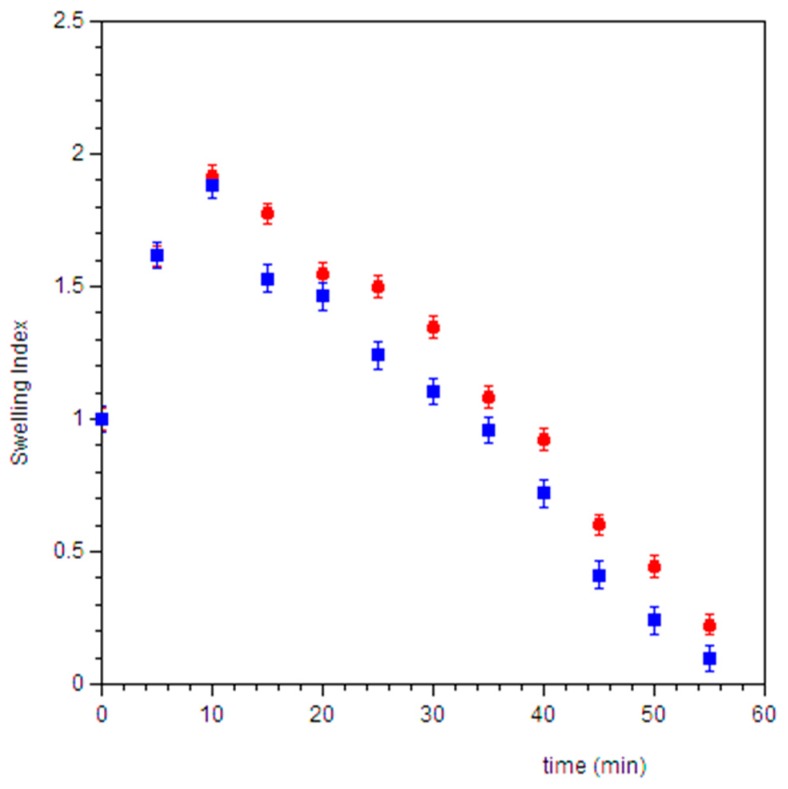
Swelling index (⬤ AFA100, ⏹ AFA300) measured as the ratio between the weight of the swollen film and dry film vs. time. Values are presented as means ± SE (n = 6).

The data related to the weight variation (Figure 8) show that the weight of the disk increases in the first 10 min-interval, due to the absorption of saliva and the consequent swelling. However, the weight of the swollen film does not reach two-fold its dry weight for both formulations, demonstrating a low swelling capacity. After this time, the weight decreases due to the dissolution of the dosage form and the release of actives. No differences were observed between AFA100 and AFA300, indicating that the residues of cells wall in AFA100 do not interfere with the uptake of saliva and the erosion process of the matrix.

### 3.7. In Vitro Dissolution Test

**Figure 9:**
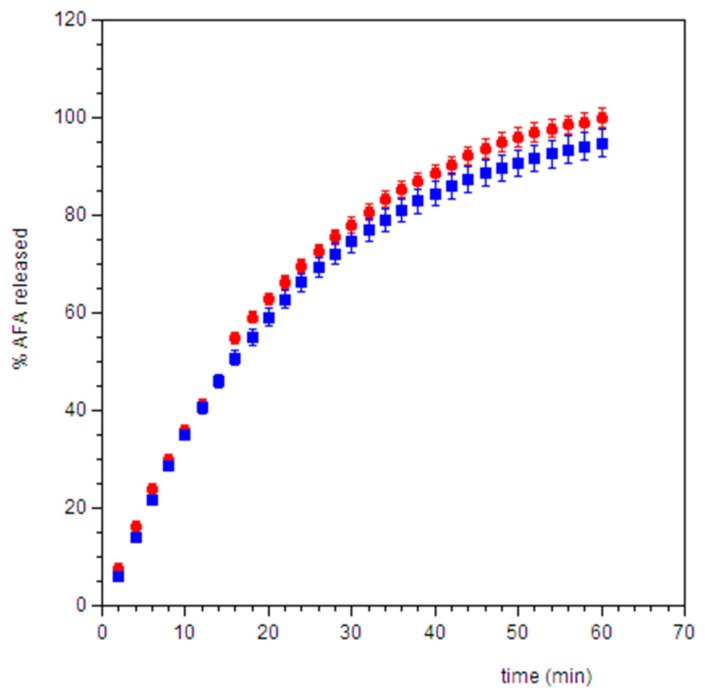
Cumulative percent of AFA released from film disks (⬤ AFA100, ⏹ AFA300) in simulated saliva pH 6.8. Values are presented as means ± SD (n = 6).

Dissolution test is a physical test capable of predicting the release of the functional substance in a certain place, in a given quantity and at a certain time. Release tests were performed using a flow through cell system able to simulate the intraoral conditions, in particular, the saliva turnover in the oral environment [65,66]. The results (Figure 9) show that, for both formulations, the 100% of AFA was released in approximately 60 min, demonstrating a functional dissolution process for our purposes.

### 3.8. Ex-Vivo Permeation Studies through Porcine Mucosae of AFA Released from Films

In order to evaluate the ability of AFA’s to activate to permeate mucosa and reach plasmatic environment, ex vivo permeation studies were performed using vertical Franz type diffusion cells. Therefore, porcine buccal and sublingual mucous membranes were chosen, due to their similarities with human epithelia in terms of lipid content, composition, morphology, and thickness [67].

The experiments were carried out by placing a 0.8 cm diameter film of AFA100 or AFA300 covered with 0.4 mL of simulated saliva in the donor compartment. As a control, experiments using a 15 mg/mL AFA solution in simulated saliva were performed. This concentration was chosen because it is comparable to the concentration obtained by the dissolution of an AFA300 film in 0.4 mL of saliva.

When AFA was applied on buccal mucosa, both administered as solution as well as films (both AFA100 and AFA300), it does not reach the acceptor fluid, up to 4 h. At the end of experiments, it was possible to observe that the films were largely dissolved. Therefore, a methanol extraction of the mucosal membrane was carried out. The results showed a tendency of AFA to accumulate into the mucosa, which increases when AFA films were applied. In Table 5, the percentage of AFA’s dose entrapped into the tissue was reported. Mucosal extraction from the control experiments showed no absorption at the wavelengths typical of AFA.

**Figure 10:**
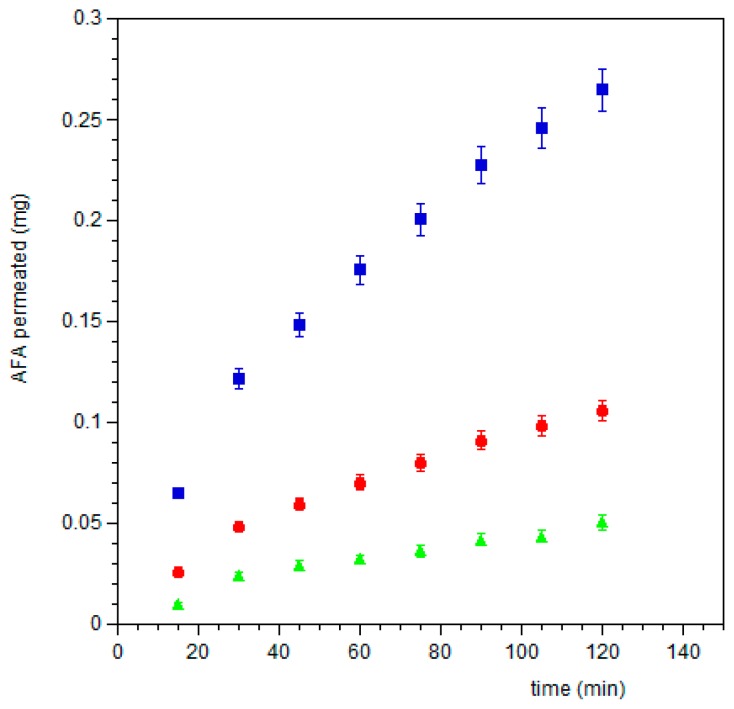
Plot of cumulative amount of AFA permeated across porcine sublingual mucosa vs. time from ⬤ AFA100 film, ⏹ AFA300 film, and ▲ solution. Values are presented as means ± SD (n = 12).

Since the sublingual route is an attractive site to deliver drugs in bloodstream bypassing the hepatic firstpass metabolic processes and giving acceptable bioavailability, the ex vivo experiments using sublingual tissue were also performed. When AFA100, AFA300 films or AFA solution were applied on sublingual mucosa AFA was able to cross it and quantitatively reach the acceptor compartment. Figure 10 shows AFA movement from the films or solution to the serosal side of sublingual tissue, expressed as the cumulative amount of permeated AFA versus time (min). It was observed that the entrapment of AFA in a matrix film promotes its permeation respect to solution and increasing AFA loaded in film means an increase of the amount of AFA that reaches the acceptor compartment.

**Figure 11:**
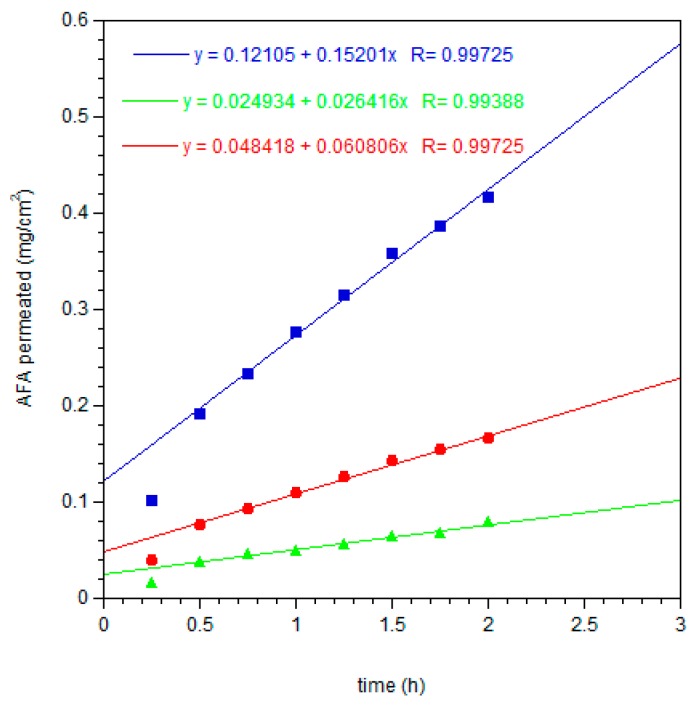
Linear fit at the steady state of AFA permeation per cm^2^ of porcine sublingual mucosa, from ⬤ AFA100 film, ⏹ AFA300 film, and ▲ solution.

Extrapolating the flux (*J*_s_) per unit area of AFA through the mucosal membrane at the steady state (Figure 11), the results demonstrated that the new dosage forms applied on porcine sublingual mucosa for two hours produced an input of AFA in the acceptor compartment with a drug flux (*J*_s_) of 0.061 ± 0.00268 mg/cm^2^·h and 0.152 ± 0.00668 mg/cm^2^·h for AFA100 and AFA300, respectively. On the other hand, the administration of 15 mg/mL solution produced a flux of 0.025 ± 0.00174 mg/cm^2^·h.

At the end of experiments, the drug’s extraction from the sublingual membranes was carried out and the results are summarized in Table 5.

It was observed that the amount of AFA entrapped in each type of mucosa is similar when films with different AFA loads were administered, probably due to a sort of membrane saturation. The greater amount of AFA detected in sublingual, rather than buccal mucosa, is probably due to the different histology of mucosal tissue, being the sublingual one, richer in water and less keratinized.

### 3.9. Stability Evaluation of Films

In order to evaluate the stability of AFA films over time, tests were conducted on AFA100 and AFA300 films packed in polyethylene bags heat-sealed and kept protected from light and room temperature for six months.

**Figure 12:**
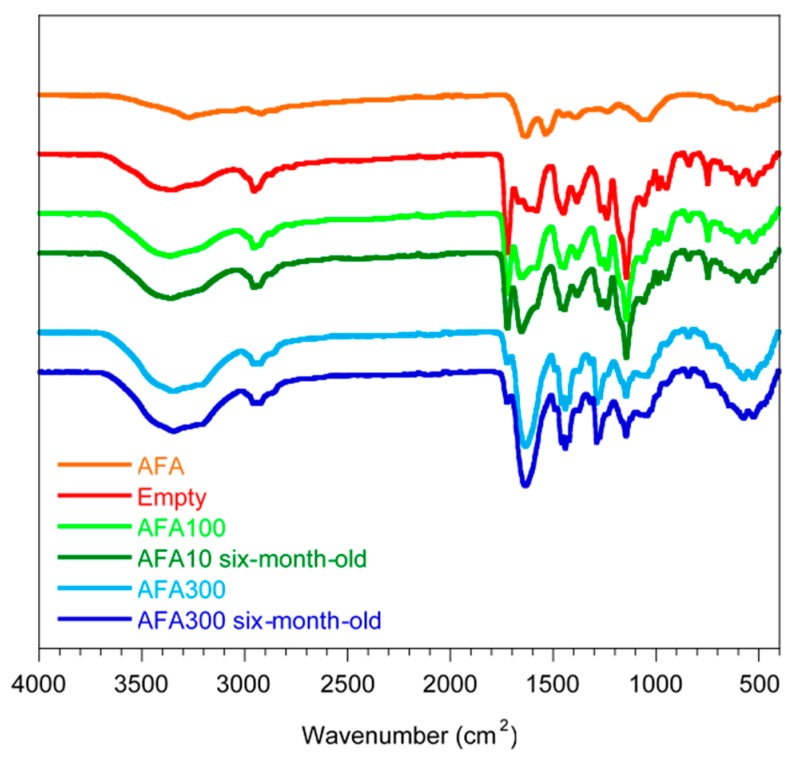
ATR-FTIR Spectra of Six-month-old AFA100 and AFA300 films compared to AFA raw material, empty film, and AFA100 and AFA300 freshly prepared.

Six-month-old AFA100 and AFA300 films were analyzed by ATR-FTIR and compared with spectra of AFA raw material, empty film, and AFA100 and AFA300 films freshly prepared (Figure 12).

Spectra of empty film, AFA100 and AFA300 show the characteristic bands of the ester groups at 1145 and 1239–1241 cm^−1^, as well as the C=O ester vibration at 1722 cm^−1^. In addition, CH_X_ vibrations can be discerned at 1386, 1450, and 2955 cm^−1^ all attributable at Eudragit E. The absorptions around 2800 cm^−1^ of dimethylamino groups do not appear, likely due to interaction with Citric acid. AFA spectrum shows three bands at 1643, 1539, and 1061 cm^−1^. The band at 1643 of AFA is identifiable as shifted at 1653 in AFA100 spectrum, and better marked at 1635 cm^−1^ in AFA300. ATR spectra were recorder also on six-month-old films. No changes in signals are highlighted, demonstrating that AFA100 and AFA300 maintain unaltered components.

Dissolution testing is an important tool for detecting formulation changes that affect the dissolution rate of a drug product. In order to assess and keep the drug release performance of films over time, an in vitro dissolution test was repeated on six-month-old AFA100 and AFA300 films.

The drug release rate from six-month-old films was negligibly slower than from the fresh ones. For instance, at 30 min after the beginning of the experiment, the percentage of AFA released from freshly AFA100 and AFA300 films, was 2.4, and 3.2%, respectively (Figure S8). The differences among the release rate are not relevant, considering the uncertainties associated with the experimental method (±5%). As each experiment was conducted in triplicate, no statistical test was applied to quantify the significance of differences.

From the overall results, several considerations could be made. The first, the matrix film promote membrane penetration of AFA both in buccal and sublingual tissue respect to a high concentrate AFA solution, indicating that new formulations could have loco-regional action by releasing antioxidants into membranes. The second, AFA300 film could be applied sublingually as a supplement to food to obtain useful plasma concentrations of antioxidants, reducing oral doses and avoiding gastro-intestinal degradation. The third, the control of AFA release could improve drug penetration allowing an accumulation in the tissue of AFA components that could be beneficial to treatment of oxidative stress-related oral diseases.

## 4. Conclusions

The reported beneficial antioxidant effects of AFA induced us to formulate and characterize a new dosage form finalized for the treatment of the OS-related oral diseases. Preliminary high frequency homogenization in ethanol of Alga demonstrated to allow an extract with high antioxidant capacity, which was successfully embedded in a matrix thin layer film. A comprehensive characterization was performed showing that the formulated films are homogeneous evidenced by weight, thickness, and drug content uniformity, and by mechanical tests that confirm a marked isotropy of material. Due to their mucoadhesiveness, the films are able to promote AFA components penetration in buccal mucosa in which they could perform the antioxidant activity.

Furthermore, if sublingually administered, the films could represent a new food supplement capable of producing high plasma levels of antioxidants. Therefore, thin layer films could be an interesting alternative for development of cosmetic and nutraceutical products with functional appeal containing plant extracts.

## Figures and Tables

**Table 1:** Film weight, thickness and drug loading of 0.125 cm^2^ disks (means ± SD).

Batch.	Weight (mg)	Thickness (mm)	AFA Content (mg)	AFA % (*w*/*w*)	AFA (mg)/cm^2^	pH Surface
AFA100-A	7.52 ± 0.09	0.604 ± 0.03	0.51 ± 0.05	6.75	4.08	6.36 ± 0.24
AFA100-B	7.39 ± 0.03	0.573 ± 0.03	0.51 ± 0.01	6.88	4.08	6.31 ± 0.22
AFA100-C	7.10 ± 0.08	0.593 ± 0.02	0.50 ± 0.04	7.05	4.00	6.45 ± 0.34
AFA300-A	7.35 ± 0.21	0.515 ± 0.01	1.36 ± 0.02	18.50	10.88	6.55 ± 0.18
AFA300-B	7.25 ± 0.31	0.551 ± 0.08	1.22 ± 0.08	16.82	9.76	6.51 ± 0.28
AFA300-C	7.56 ± 0.30	0.485 ± 0.07	1.33 ± 0.10	17.59	10.64	6.35 ±0.14

**Table 2:** Values obtained from Uniaxial tests on AFA100 and AFA300: Average ± SD.

Film Composition	*σ*_u_ along x_1_ (MPa)	*σ*_u_ along x_2_ (MPa)	*ε*_u_ along x_1_ (%)	*ε*_u_ along x_2_ (%)	*E*_1_ (Mpa)	*E*_2_ (Mpa)
AFA100	0.11976 ± 0.03563	0.10987 ± 0.03711	50.013 ± 0.05012	45.120 ± 0.04415	5.08329 ± 0.00331	5.10010 ± 0.00103
AFA300	0.12512 ± 0.03751	0.11216 ± 0.03824	51.117 ± 0.07310	47.209 ± 0.05419	5.09335 ± 0.00376	5.15611 ± 0.00208

**Table 3:** Mean value (±SD) of mechanical parameters with biaxial tests on AFA films.

AFA100	AFA100	AFA300	AFA300
$\nu_{12}$	$\nu_{21}$	$\nu_{12}$	$\nu_{21}$
0.42935 ± 0.27668	0.42832 ± 0.18837	0.42283 ± 0.28361	0.42202 ± 0.20014
$E_{1}$ (MPa)	$E_{2}$ (MPa)	$E_{1}$ (MPa)	$E_{2}$ (MPa)
5.00210 ± 0.33762	5.09913 ± 0.29961	5.00691 ± 0.28890	5.09911 ± 0.29157

**Table 4:** Force of adhesion and Detachment Force of matrix Films as function of time contact with mucosa. Values are presented as means ± SE.

Time of Contact	Formulation	Force of Adhesion ± SE (N)	Detachment Force (N/m^2^)
5 min	AFA100	0.0592 ± 0.001	1178.34
	AFA300	0.0595 ± 0.008	1184.58
10 min	AFA100	0.0772 ± 0.011	1536.62
	AFA300	0.0621 ± 0.010	1235.98
15 min	AFA100	0.0743 ± 0.007	1478.90
	AFA300	0.0648 ± 0.011	1290.00

**Table 5:** Results of AFA entrapment in buccal and sublingual mucosae after administration of solution and films.

Sample	Administered AFA Dose	AFA (mg) ± SE into Buccal Mucosa after 4 h	% of Dose into Buccal Mucosa after 4 h	AFA (mg) ± SE into Sublingual Mucosa after 2 h	% of Dose into Sublingual Mucosa after 2 h
Solution	15 mg/mL in saliva	0.361 ± 0.074	2.40	0.535 ± 0.087	3.56
AFA100	2.4 ± 0.4 mg in 0.4 mL saliva	0.449 ± 0.181	18.70	0.612 ± 0.071	25.50
AFA300	6.5 ± 0.5 mg in 0.4 mL saliva	0.455 ± 0.093	8.24	0.648 ± 0.086	11.52

## Supplementary Materials

The following are available online at http://www.mdpi.com/1999-4923/11/1/35/s1, Figure S1. 3D image of AFA methanol extract elution from 0 to 30 min. Figure S2. (a) Chromatographic elution of AFA methanol extract with UV wavelength set at 407 nm from 0 to 30 min. (b) Enlargement from 0 to 8 min of chromatographic elution. Figure S3. Chromatographic elution of AFA methanol extract with UV wavelength set at 334 nm from 0 to 30 min. Figure S4. Chromatographic elution of AFA methanol extract with UV wavelength set at 260 nm from 0 to 30 min. Figure S5. 3D image of AFA ethanol extract elution from 0 to 30 min. Figure S6. Chromatographic elution of AFA ethanol extract with UV wavelength set at 407 nm from 0 to 30 min. Figure S7. Chromatographic elution of AFA ethanol extract with UV wavelength set at 334 nm from 0 to 30 min. Figure S8. Cumulative percent of AFA released from film disks (⬤ AFA100, ⏹ AFA300, ▲ AFA100 six-month old, ▼ AFA300 six-month old) in simulated saliva pH 6.8.
