# Supplementary material for: Enhanced In Situ Availability of Aphanizomenon Flos-Aquae Constituents Entrapped in Buccal Films for the Treatment of Oxidative Stress-Related Oral Diseases: Biomechanical Characterization and In Vitro/Ex Vivo Evaluation
Source: Pharmaceutics. 2019 Jan 17;11(1):35. doi: 10.3390/pharmaceutics11010035 (PMC6359436; doi:10.3390/pharmaceutics11010035)
Supplement: Supplementary file 1 [file pharmaceutics-11-00035-s001.pdf]

pharmaceutics

Supplementary Materials: Enhanced In Situ Availability of *Aphanizomenon Flos-Aquae* Constituents Entrapped in Buccal Films for the Treatment of Oxidative Stress-Related Oral Diseases: Biomechanical Characterization and In Vitro/Ex Vivo Evaluation

Viviana De Caro, Denise Murgia, Francesco Seidita, Emanuela Bologna, Gioacchino Alotta, Massimiliano Zingales and Giuseppina Campisi

HPLC analysis of AFA

HPLC analyses were performed with a HPLC Shimadzu LC-10AD VP instrument equipped with a binary pump LC-10AD VP, a UV SPD-M20A Diode Array detector, a 20µL injector and a computer integrating apparatus (EZ Start 7.4 software). Chromatographic separation was achieved on a reversed-phase column SeQuant® Zic®-Hilic, (5 µm, 200 Å,150 × 2.1 mm), a mobile phase consisted of acetate buffer 5 mM pH 6.5 (A) and Acetonitrile (B). For separation of AFA components the gradient method was developed as follows: A:B ((0.5:99.5→0.01–5.00 min, 40:60→5.00–11.00 min; 40:60→11.00–30.00 min). The flow rate was set at 0.3 mL/min, the UV wavelength range 200–700 nm and set at 260, 334, 407, 665 nm to identification.

In these conditions, the Methanol AFA extract obtained by high frequency homogenization, showed the retention time (Rt) for PCs at 1.79 and 1.98 min, whereas for MAAs at 20.47 min. Following is shown in figure S1 the 3D image of the chromatographic elution in order to highlight all the separated compounds that have different maximum absorption peaks.

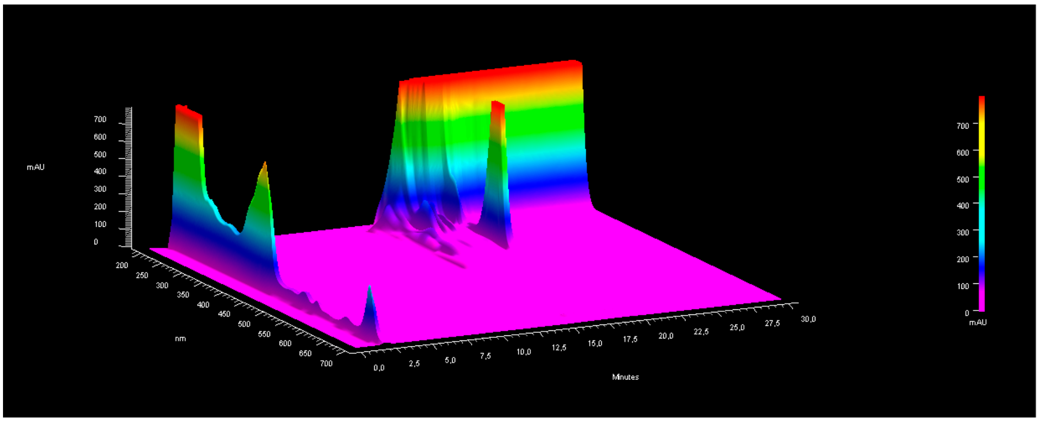

Figure S1. 3D image of AFA methanol extract elution from 0 to 30 min.

Following is shown (Figure S2) the chromatogram with the detector set at a wavelength of 407 nm. The picks at Rt 1.79 and 1.98 min were attributed to PCs.

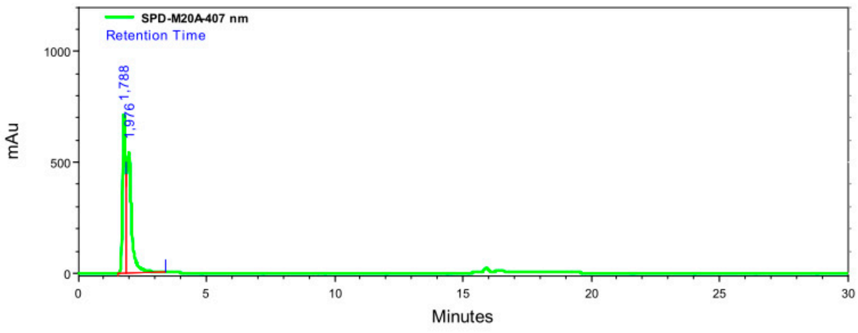

(a)

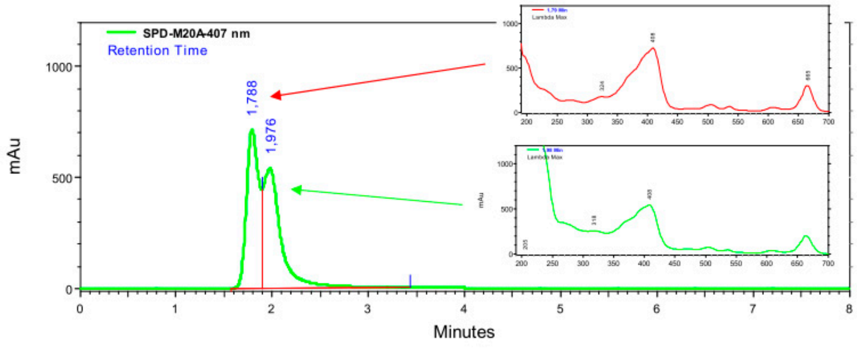

(b)

Figure S2. (**a**) Chromatographic elution of AFA methanol extract with UV wavelength set at 407 nm from 0 to 30 min. (**b**) Enlargement from 0 to 8 min of chromatographic elution.

Following is shown (Figure S3) the chromatogram with the detector set at a wavelength of 334 nm. The pick at Rt 20.47 min was attributed to MAAs (probably Porphyra-334).

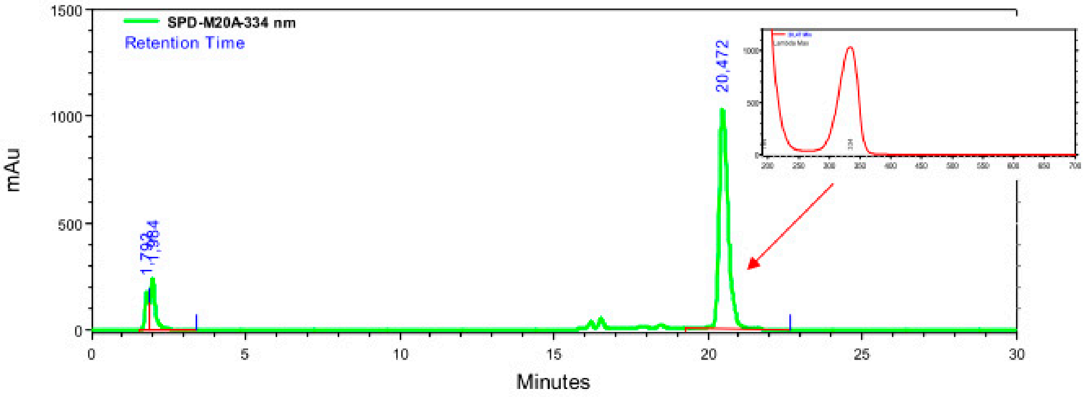

Figure S3. Chromatographic elution of AFA methanol extract with UV wavelength set at 334 nm from 0 to 30 min.

Following is shown (Figure S4) the chromatogram with the detector set at a wavelength of 260 nm. The picks at Rt ranged 16.5 to 20.0 min were attributed to phenolic components.

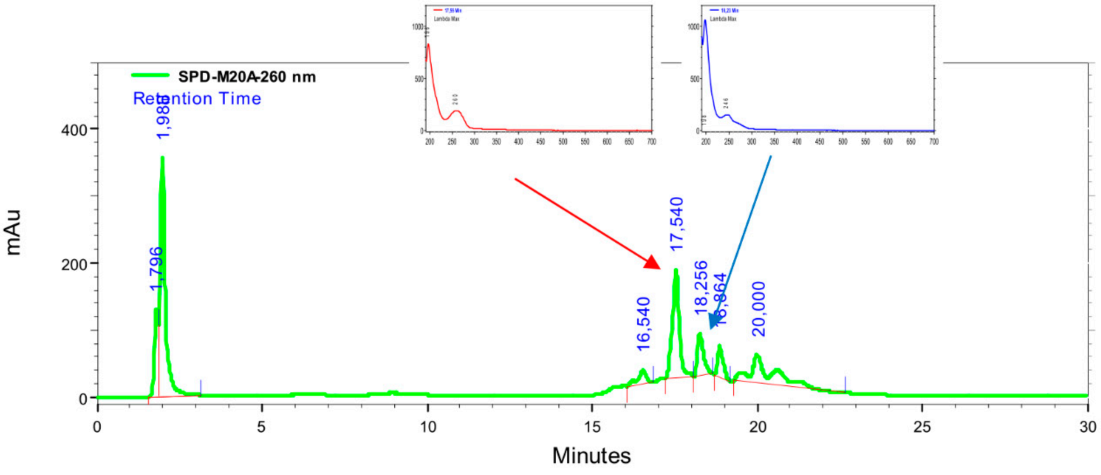

Figure S4. Chromatographic elution of AFA methanol extract with UV wavelength set at 260 nm from 0 to 30 min.

In the conditions above mentioned, the Ethanol AFA extract obtained by high frequency homogenization, showed the retention time (Rt) for PCs at 1.47 and 1.89 min, whereas for MAAs at 2.76 and 3.38 min. No peak was observed around 20 min of elution. Following is shown in figure S5 the 3D image of the chromatographic elution in order to highlight all the separated compounds that have different maximum absorption peaks.

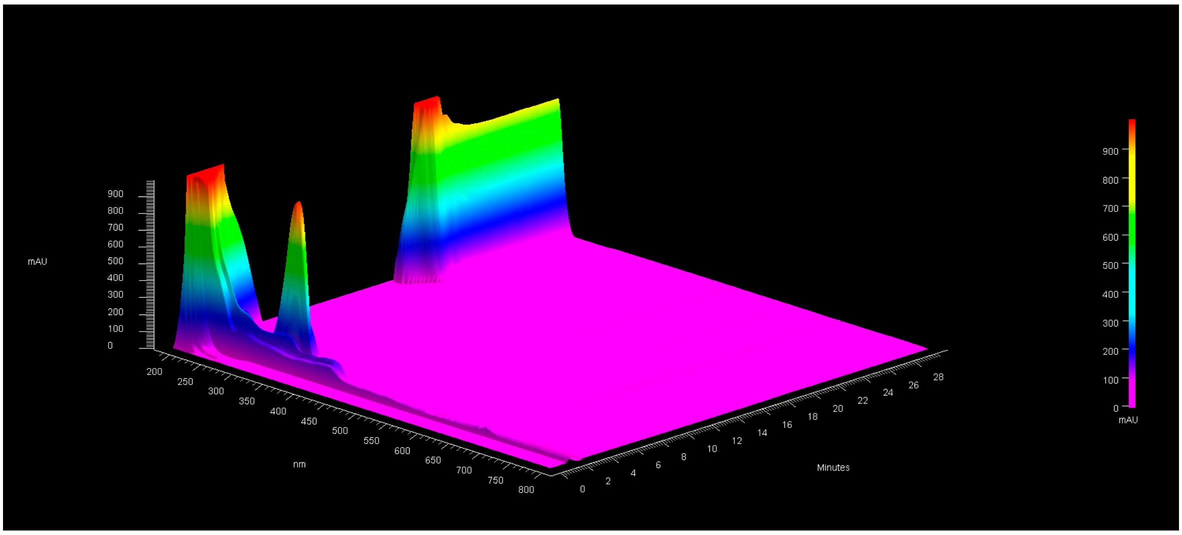

Figure S5. 3D image of AFA ethanol extract elution from 0 to 30 min.

Following is shown (figureS6) the chromatogram with the detector set at a wavelength of 409 nm. The picks at Rt 1.47 and 1.89 min were attributed to PCs.

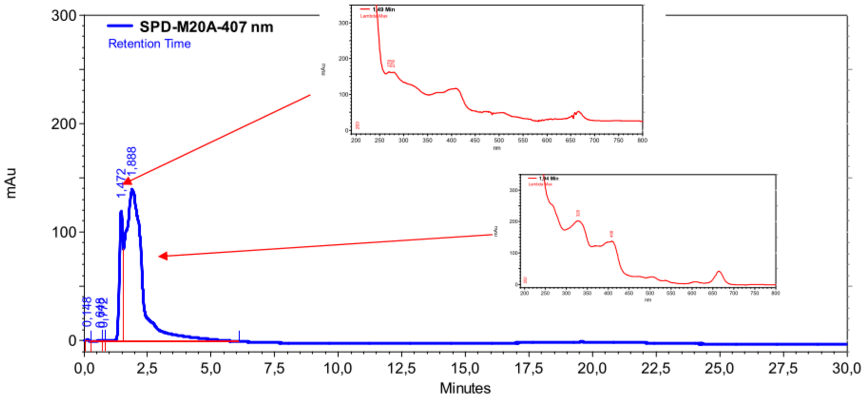

Figure S6. Chromatographic elution of AFA ethanol extract with UV wavelength set at 407 nm from 0 to 30 min.

Following is shown (figure S7) the chromatogram with the detector set at a wavelength of 334 nm. Two picks at Rt 2.76 and 3.38 min were attributed to MAAs.

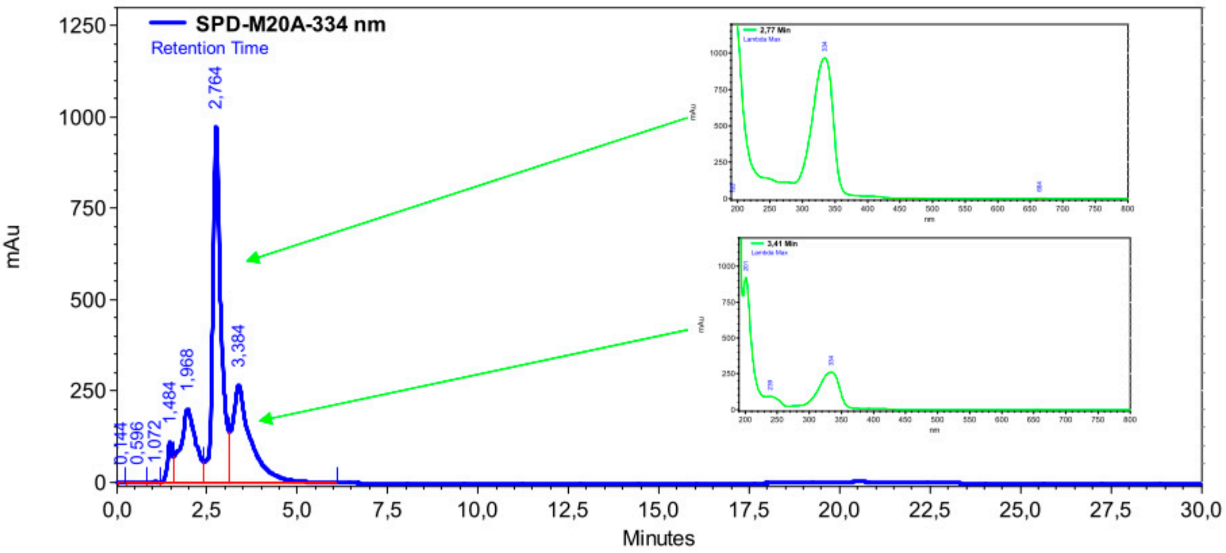

Figure S7. Chromatographic elution of AFA ethanol extract with UV wavelength set at 334 nm from 0 to 30 min.

Dissolution test

The results of dissolution test of AFA100 and AFA300 films freshly prepared were compared to those obtained from six-month-old AFA100 and AFA300 films (Figure S8).

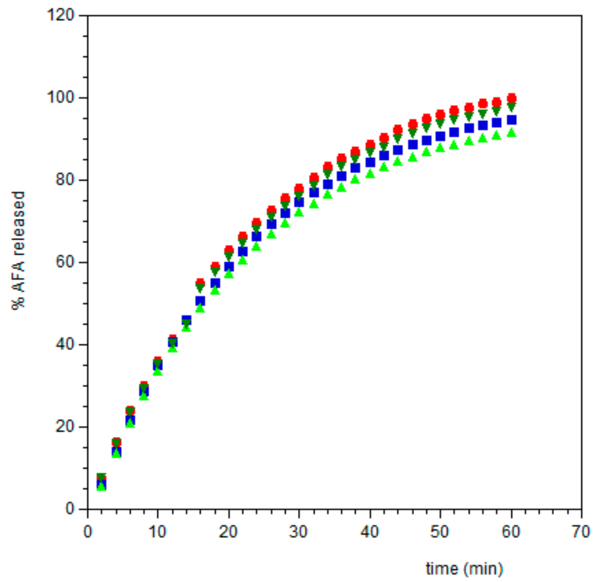

Figure S8. Cumulative percent of AFA released from film disks (● AFA100, ■ AFA300, ▲ AFA100 six-month old, ▼ AFA300 six-month old) in simulated saliva pH 6.8.

The drug release rate from six-month old films was negligibly slower than from the freshly ones. For instance, at 30 min after the beginning of the experiment, the percentage of AFA released from freshly AFA100 and AFA300 films, was 2.4, and 3.2%, respectively. The differences among the release rate are not relevant, considering the uncertainties associated with the experimental method (± 5%). As each experiment was conducted in triplicate, no statistical test was applied to quantify the significance of differences.
